# Supplementary material for: Ixazomib, Lenalidomide, and Dexamethasone (IRD) Treatment with Cytogenetic Risk-Based Maintenance in Transplant-Eligible Myeloma: A Phase 2 Multicenter Study by the Nordic Myeloma Study Group
Source: Cancers (Basel). 2024 Feb 29;16(5):1024. doi: 10.3390/cancers16051024 (PMC10930875; doi:10.3390/cancers16051024)
Supplement: Supplementary file 1 [file cancers-16-01024-s001.zip › cancers-2869496-SI.pdf]

Supplement figure S1. Progression-free survival according to detailed risk groups (ITT)

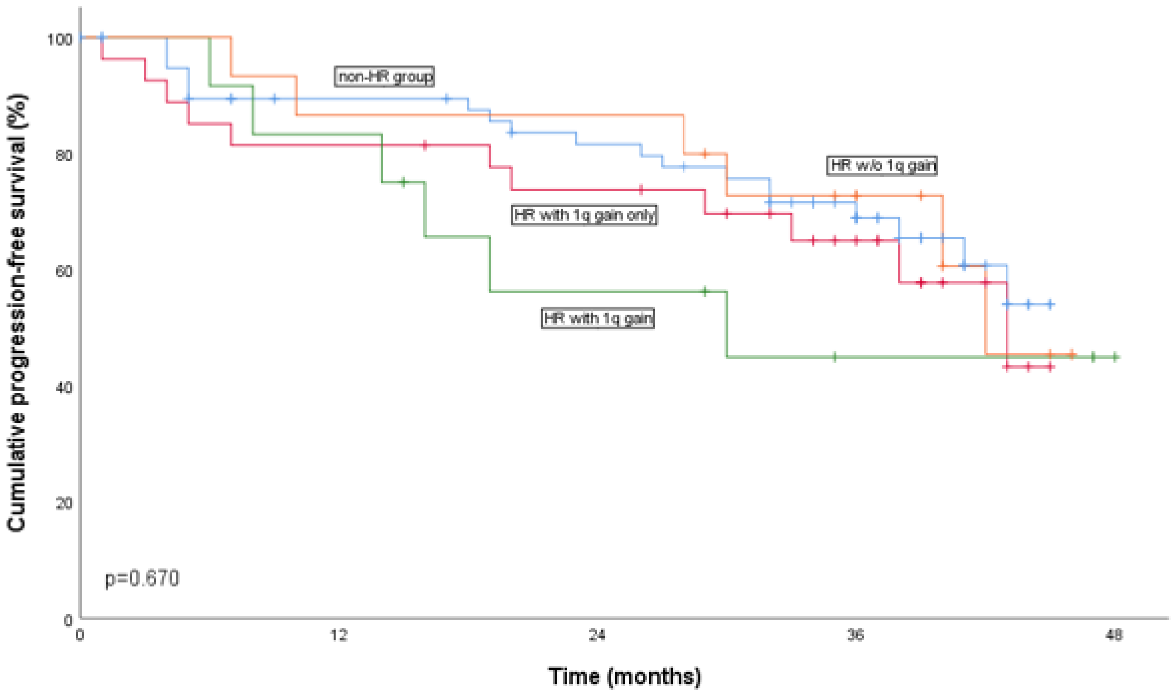

Patients at risk

62	47	41	26	0
29	22	19	11	0
11	10	6	3	0
14	13	12	7	0

Supplement figure S2. Progression-free survival a) and overall survival b) in myeloma patients with sustained undetectable MRD $<10^{-5}$

a.

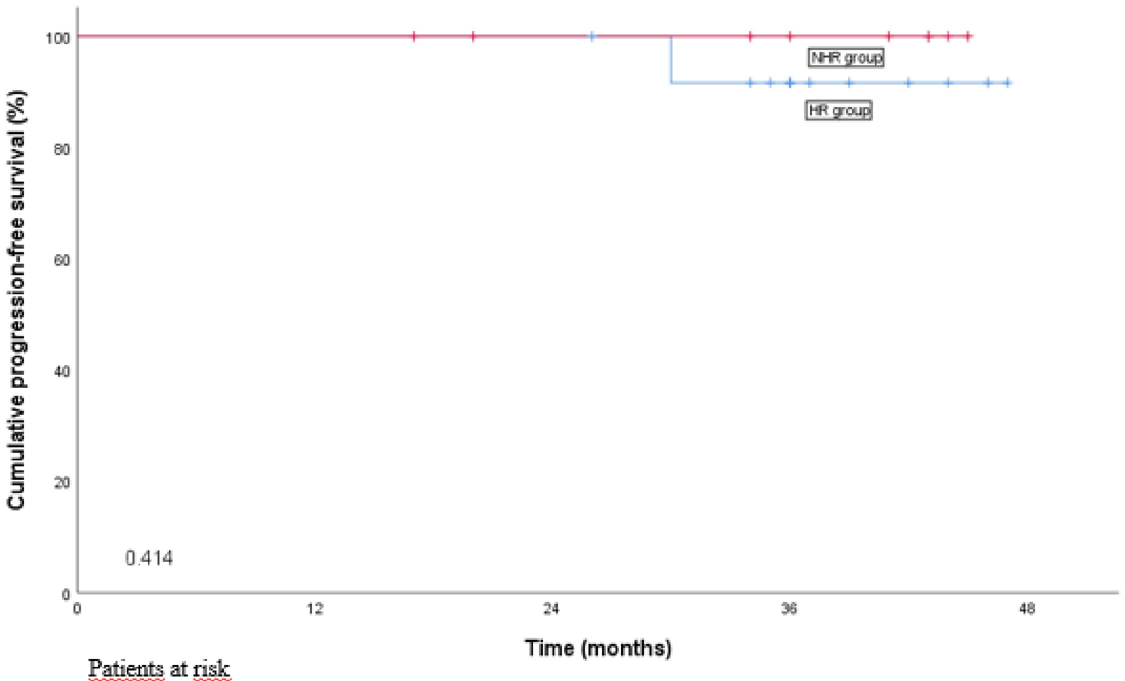

Patients at risk

13	12	12	8	0
10	9	8	6	1

b.

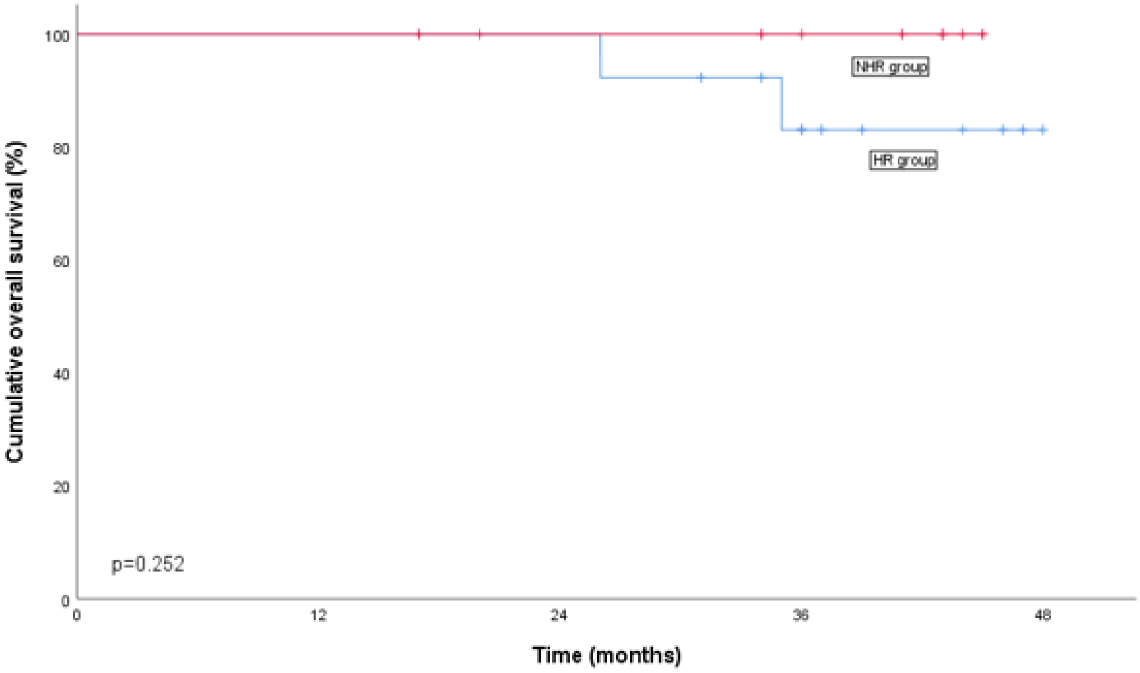

Patients at risk

13	12	12	8	0
10	9	8	6	1

**Supplement Table S1. Mobilization and collection data of 108 myeloma patients according to the risk groups**			
**Variable**	**HR group n=54 (%)**	**NHR group n=54 (%)**	**Significance *p***
**Mobilization type**			0.272
**CY+FIL**	37 (68)	43 (80)	
**CY+PEG**	1 (2)		
**FIL alone**	16 (30)	11 (20)	
**PLER use**	20 (35)	14 (22)	0.270
**Peak blood $CD34^+$ cell count (x 10^6/L), median (range)**	42 (13-106)	77 (16-212)	**0.045**
**Total collected $CD34^+$ cell yield † (x 10^6/kg), median (range)**	5.6 (0.2-19.2)	6.8 (1.8-15.9)	0.119
**Number of apheresis**			0.389
**1**	23 (43)	23 (43)	
**2**	19 (35)	24 (44)	
**3**	6 (11)	4 (7)	
**4**	5 (9)	1 (2)	

Abbreviations: CY+FIL=cyclophosphamide+filgrastim; CY+PEG=cyclophosphamide+pegfilgrastim; FIL=filgrastim;PLER=plerixafor; HR=high risk
† four mobilization failures

Supplement Table S2. Associations of different variables with PFS in 120 patients with myeloma			
**Variable**	**Exp (B)**	**95% CI**	***p***
**Gender**			
**Male**	1		
**Female**	1.648	0.899-3.022	0.106
**Age**			
**≤65**	1		
**>65**	1.464	0.756-2.833	0.258
**ECOG**			
**0**	1		
**1**	1.076	0.561-2.063	0.826
**2**	1.150	0.391-3.383	0.799
**IMWG risk group**			
**low**	1		
**standard**	2.384	0.842-6.749	0.102
**high**	1.654	0.464-5.895	0.437
**R-ISS**			
**1**	1		
**2**	2.656	1.163-6.065	**0.020**
**3**	7.279	2.415-21.940	< **0.001**
**Cytogenetic risk group**			
**HR**	1		
**NHR**	0.770	0.419-1.415	0.400
**Best serological response**			
**sCR**	1		
**CR**	0.933	0.503-1.733	0.827
**VGPR**	0.854	0.452-1.614	0.628
**PR**	1.328	0.608-2.899	0.477
**Flow-MRD 10^{-4}**			
**sustained undetectable**	1		
**turned positive**	40.382	5.039-323.625	**< 0.001**
**positive**	28.167	3.843-206.453	**0.001**
**undetectable once**			0.981
**Flow-MRD 10^{-5}**			
**sustained undetectable**	1		
**turned positive**	11.106	1.300-95.571	**0.028**
**positive**	16.495	2.256-120.596	**0.006**
**undetectable once**			0.982
